# When Is Moving a Cursor With a Computer Mouse Intuitive?

**DOI:** 10.1177/0301006620915152

**Published:** 2020-04-01

**Authors:** Eli Brenner, Myriam L. de Graaf, Marielle J. Stam, Margreet Schonwetter, Jeroen B. J. Smeets, Robert J. van Beers

**Affiliations:** Department of Human Movement Sciences, Vrije Universiteit Amsterdam, Amsterdam, The Netherlands; Department of Human Movement Sciences, Vrije Universiteit Amsterdam, Amsterdam, The Netherlands; Department of Movement Science, University of Münster, Germany; Department of Human Movement Sciences, Vrije Universiteit Amsterdam, Amsterdam, The Netherlands

**Keywords:** perception/action, frames of reference, computer mouse, tool use, sensorimotor transformations

## Abstract

People have a good intuition of how to move a computer mouse to place a cursor at a desired position on a screen. This is surprising because the hand and the mouse are at different locations and they generally move in different directions and over different distances. But using a computer mouse is not always intuitive: try positioning a cursor after turning the mouse by 90° in your hand. To examine when using a computer mouse is intuitive, we asked participants to move a cursor to targets on a screen by moving a mouse along a surface. We varied the orientation of this surface in space and that of the mouse in the hand. Participants performed best when the mapping between hand and cursor motion was close to what we are accustomed to, either in space or relative to the forearm.

We asked participants to quickly move a cursor to a target on a vertical computer screen (38 × 30 cm) by moving a wireless computer mouse. They moved the mouse across a surface that was attached to a sturdy adjustable tripod. When the cursor (a 1.2-cm diameter black dot) remained within the target (a 3.5-cm diameter green dot) for 200 ms, the target jumped to a new position (9 cm away in a randomly selected direction). This continued for 90 s. We compared 13 configurations that only differed in where the mouse was held and the direction in which it had to be moved to move the cursor in a desired direction. Eighteen right-handed adult participants signed informed consent forms, practiced in the normal configuration, and were then tested once in each configuration in random order.

The medians of the times taken to reach targets were ranked across configurations for each participant individually. Almost all participants reached targets fastest in the normal configuration with the mouse at hip level in front of their right shoulder (Figure 1A). It consistently took them longer to reach targets when the arm with the mouse pointed to their right, despite this completely changing the mapping between the directions of motion of the cursor and of the hand in space (Figure 1B). Despite the consistency, it only took them slightly longer. Rotating the mouse leftwards relative to the hand to compensate for the changed mapping in space when the arm pointed to the right made it take slightly longer rather than shorter (Figure 1F), so moving along the forearm (extending the arm) seems to intuitively be mapped to displacing the cursor up the screen. Indeed, it also took a similar amount of time to reach targets when participants moved the mouse downwards along a vertical surface aligned with their body (Figure 1C), moved it to the left while the hand holding the mouse was oriented to the left (Figure 1D), or moved the mouse forwards against a vertical surface that was orthogonal to their body (like a wall on their right) with their elbow bent and their whole arm approximately at shoulder level (Figure 1E). It took only slightly longer when the mouse was held upside down against the underside of a horizontal surface and its left and right coordinates were flipped so that moving the rotated forearm to the left moved the cursor to the left (Figure 1G).

**Figure 1:**
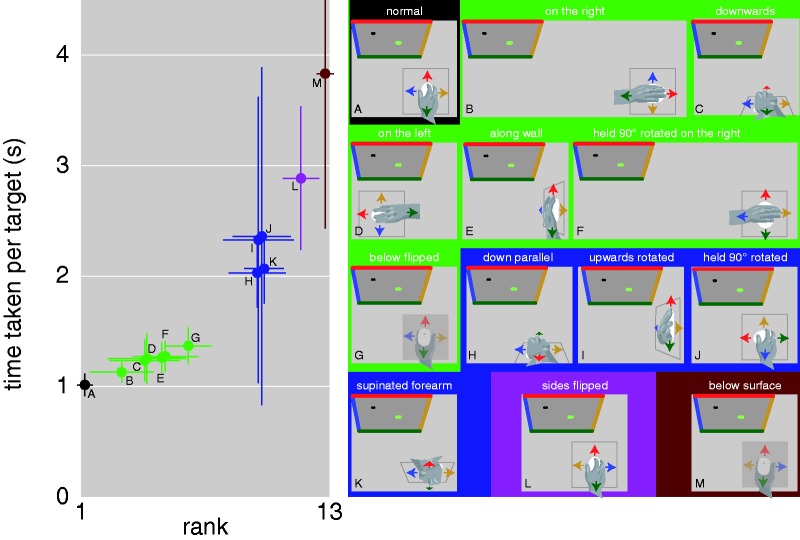
Median Time Taken to Reach the Target in Each Configuration. Symbols show means and standard deviations across participants, both for the median times taken per participant and their rank orders. The schematic drawings illustrate top views of the various configurations (A–M). Colored arrows indicate directions of motion that will make the cursor move in the corresponding direction on the screen. Symbol colors and backgrounds of the schematic drawings indicate groups of configurations that we consider to have similar difficulty.

Four configurations in which extending the arm did not move the cursor upwards took about twice as long per target. In two of these configurations, the hand moved parallel to the cursor, either along a vertical surface as in Figure 1C but with the up and down coordinates flipped (Figure 1H), or along the back of the same surface with the forearm rotated and the mouse coordinates rotated by 180° with respect to normal (Figure 1K). The other two configurations were ones in which the mouse was rotated in the hand as in Figure 1F, but in which it was either held at the normal position in front of the right shoulder (Figure 1J), or moved along the “wall” (as described for Figure 1E) while being held with the elbow below the hand (Figure 1I). Finally, there were two configurations that were consistently even more difficult to cope with. In both cases moving to the left made the cursor move to the right, and vice versa. In one case this was because the mouse directions were flipped (Figure 1L) and in the other because the mouse was held upside down against a surface from below (Figure 1M).

When the mouse was held upside down below a surface as in Figure 1M, with the palm of the hand facing upwards so that the thumb was on the right rather than on the left, flipping the lateral mapping of the mouse as in Figure 1L made the task much easier (Figure 1G), indicating that movement directions in space are relevant. However, the orientation of the arm is clearly also relevant, because performance was similar for very different mappings between hand and cursor in space as long as the cursor responded in the same way to similar movements relative to the arm (Figure 1A to E). Moreover, the exact same movement of the mouse in space was treated differently when the arm was held differently (compare Figure 1E and I). In the usual and most comfortable (Wigdor et al., 2006) configuration, the movement plane is perpendicular to the screen. Placing the screen horizontally so that the cursor moves parallel to the hand is known to make guiding the cursor more difficult rather than easier (Pavlovych & Stuerzlinger, 2008). Here, rotating the movement plane so that the cursor moved parallel to the hand (Figure 1H and K) doubled the time per target. Performance in these configurations did not improve more between the first and last five trials than in other configurations with similar overall performance (Figure 1I and J), so it is probably not just a matter of practice (although practice does improve performance; Cunningham, 1989). In one configuration (Figure 1F), performance was quite good although moving along the forearm did not move the cursor up. Maybe people rely on the usual directions in space when arm-based directions fail. This is less effective when there is a 90° rotation around a vertical (Figure 1J), lateral (Figure 1H and K), or sagittal (Figure 1I) axis from the normal configuration. The task is even more difficult if more than a 90° rotation is needed to reach the normal configuration (Figure 1L and M). It would appear that using the mouse is intuitive as long as the mapping either in space or relative to the forearm is close to what we are accustomed to.
